# ReMiND: Recovery of missing neuroimaging using diffusion models with application to Alzheimer’s disease

**DOI:** 10.1162/imag_a_00323

**Published:** 2024-10-22

**Authors:** Chenxi Yuan, Jinhao Duan, Kaidi Xu, Nicholas J. Tustison, Rebecca A. Hubbard, Kristin A. Linn

**Affiliations:** Department of Biostatistics, Epidemiology & Informatics, Perelman School of Medicine, University of Pennsylvania, Philadelphia, PA, United States; Penn Statistics in Imaging and Visualization Center, Perelman School of Medicine, University of Pennsylvania, Philadelphia, PA, United States; Department of Computer Science, College of Computing & Informatics, Drexel University, Philadelphia, PA, United States; Department of Radiology and Medical Imaging, School of Medicine, University of Virginia, Charlottesville, VA, United States

**Keywords:** diffusion model, missing image imputation, longitudinal study, magnetic resonance imaging, Alzheimer’s disease

## Abstract

Missing data is a significant challenge in medical research. In longitudinal studies of Alzheimer’s disease (AD) where structural magnetic resonance imaging (MRI) is collected from individuals at multiple time points, participants may miss a study visit or drop out. Additionally, technical issues such as participant motion in the scanner may result in unusable imaging data at designated visits. Such missing data may hinder the development of high-quality imaging-based biomarkers. To address the problem of missing MRI data in studies of AD, we introduced a novel 3D diffusion model specifically designed for imputing missing structural MRI (Recovery of Missing Neuroimaging using Diffusion models (ReMiND)). The model generates a whole-brain image conditional on a single structural MRI observed at a past visit or conditional on one past and one future observed structural MRI relative to the missing observation. The performance of models was compared with two alternative imputation approaches: forward filling and image generation using variational autoencoders. Experimental results show that our method can generate 3D structural MRI with high similarity to ground-truth images at designated visits. Furthermore, images generated using ReMiND show relatively lower differences in volume estimation between the imputed and observed images compared to images generated by forward filling or autoencoders. Additionally, ReMiND provides more accurate estimated rates of atrophy over time in important anatomical brain regions than the two comparator methods. Our 3D diffusion model can impute missing structural MRI data at a single designated visit and outperforms alternative methods for imputing whole-brain images that are missing from longitudinal trajectories.

## 1 Introduction

Alzheimer’s disease (AD) is a progressive neurodegenerative disorder characterized by a decline in cognitive abilities, including memory, language, and problem-solving abilities (De Strooper & Karran, 2016). The accurate prediction of progression from normal cognition to mild cognitive impairment (MCI) and subsequently to AD will become increasingly important for patient care and resource allocation as early interventions and treatments for the disease are developed (Aviles-Rivero et al., 2022). Brain imaging techniques, such as magnetic resonance imaging (MRI), can provide information about changes in brain structure or function that occur as AD progresses (van Oostveen & de Lange, 2021). MRI feature-based classification and prediction algorithms have a high potential for early detection of characteristic AD patterns in brain structure and activity (Arnold et al., 2011). However, in research studies that use repeated longitudinal imaging to measure brain changes over time, there is a high risk of missing data due to the susceptibility of older participants to physical and cognitive decline, illness, and death (Hardy et al., 2009). The planned imaging scans may be missing due to participant dropout, technical issues during image acquisition, or participant unwillingness to undergo imaging, resulting in the absence or incompleteness of imaging data trajectories for some study participants (Campos et al., 2015;Lo & Jagust, 2012).

The presence of missing data poses several challenges for longitudinal studies of AD, such as reducing the sample size overall or disproportionately in the AD-affected group, introducing selection bias, and reducing statistical power for estimating and evaluating the effect of imaging biomarkers (Ibrahim et al., 2012). Thus, validity and power can both be significantly impacted by the effects of missing imaging data. Researchers in AD have made efforts to impute longitudinal missing data by applying various techniques, such as forward filling, linear filling, multiple imputation, K-Nearest Neighbor, multiple kernel learning, long short-term memory, and recurrent neural networks (Campos et al., 2015;N.-H. Ho et al., 2022;Junninen et al., 2004;Lipton et al., 2016;Nguyen et al., 2020;Shishegar et al., 2021;Zhu et al., 2017). The majority of existing methods focused on generating image-derived phenotypes (IDPs) rather than imputing the entire missing image. In this work, we employ a diffusion model to generate an entire 3D image conditional on one or more observed images from an individual’s imaging trajectory.

The denoising diffusion probabilistic model (DDPM or diffusion model for short) (J. Ho et al., 2020) is a new class of generative models that utilizes a latent variable framework to reverse a diffusion process, wherein Gaussian noise is gradually added to the observed data to transform its distribution to a distribution of noise. Diffusion models have demonstrated exceptional performance in various tasks such as image, audio, and graph production, as well as conditional generation tasks such as in-painting, super-resolution, and picture editing (J. Ho et al., 2022;Kong et al., 2021;Lugmayr et al., 2022;Peng et al., 2022;Pinaya et al., 2022;Wolleb et al., 2022;Xie & Li, 2022). The diffusion models are well suited to our longitudinal imputation problem in two respects. First, the diffusion model has the ability to generate images in the temporal dimension, such as video-related tasks that predict the future frame conditioned on the past frame (Voleti et al., 2022). Longitudinal MRI image imputation is analogous to image generation in the temporal dimension. Second, the diffusion model shows promising results when synthesizing non-medical images and has rivaled state-of-the-art models such as generative adversarial nets (GANs) and variational autoencoders (Goodfellow et al., 2020;Kingma & Welling, 2013). The promise of diffusion models is due to a probabilistic process that learns biological changes over time from a diverse array of brain images, encompassing both healthy and pathological states. The probabilistic process ensures the synthesized images reflect realistic biological transitions in brain structure for a given historical image or set of images from a patient’s longitudinal trajectory (Dhariwal & Nichol, 2021). In contrast, GANs might produce modifications in images that are overly sharp or lack realism, particularly in the context of texture and edge delineation (Isola et al., 2017). This is because the adversarial process can lead to the generator producing sharp details that exploit the discriminator’s weaknesses, resulting in unrealistic textures and edges (Shamsolmoali et al., 2021). The probabilistic approach of diffusion model ensures that the generated images retain the statistical properties of the training data, leading to more natural and realistic images (Yang et al., 2023).

In this study, we have developed a novel approach called ReMiND (Recovery of Missing Neuroimaging with Diffusion models) for 3D MRI imputation in longitudinal studies of AD. ReMiND focuses on generating missing images at a designated single visit by conditioning on one or more observed images from other time points. From a model design perspective, image generation conditioned on time involves training the model using concatenated tensors of past, present, and/or future visits to minimize the generative loss based on the specified temporal conditions. The overall pipeline of the proposed ReMiND approach is illustrated inFigure 1. The main contributions of this research are as follows:

**Fig. 1.**
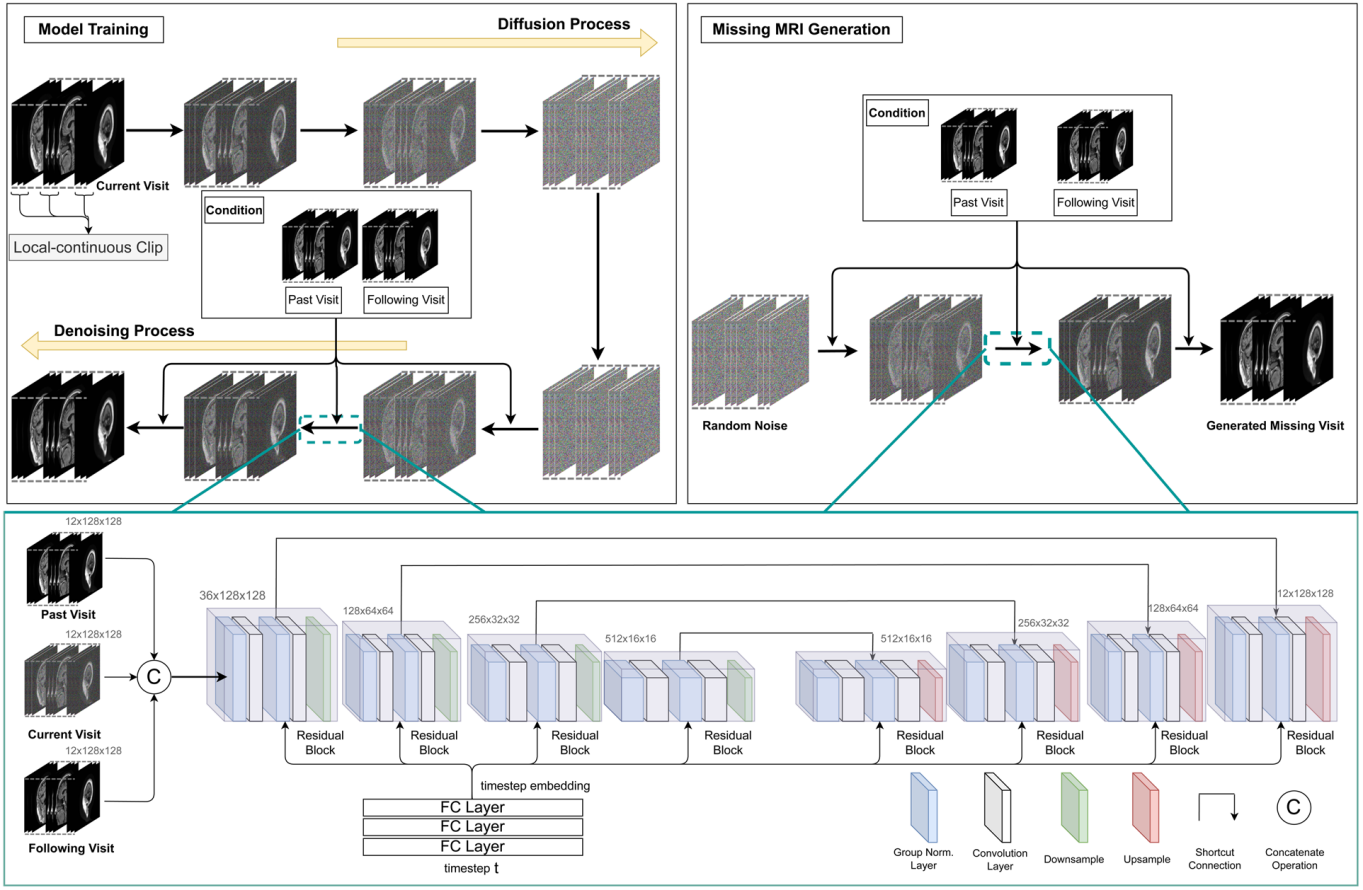
Pipeline of the proposed ReMiND model. For model training, ReMiND follows the diffusion process of a DDPM by adding random noise on a designated MRI. Then, ReMiND leverages parameterized neural networks during the denoising process to recover the noise applied, with conditions over past visits or past and following visits. To generate a missing MRI after the model is trained, ReMiND passes random noise through the learned denoising process along with one or more observed images from other time points in a subject’s longitudinal image trajectory. The denoising process is parameterized by UNet-like neural networks, taking the concatenation of conditions and intermediate results as input and predicting the added noise. FC Layer refers to Fully-Connected Layer. Group Norm refers to Group Normalization, which first aggregates activations into groups by channels and then calculates group-wise normalization. Shortcut connection refers to a branch where the source of the connection will be directly added to the end, which benefits multi-scale modeling and gradient propagation. Residual block means there will be one shortcut connection within this block. A comprehensive introduction of the network is provided in theSupplementary Material.

1. Unlike previous studies that utilized multiple imaging modalities to impute missing imaging data, our work focuses on imputing missing MRI images in the temporal dimension using images from the same modality at other time points, specifically in the context of AD.
2. We have developed a novel 3D diffusion model specifically designed for MRI image generation. The model effectively preserves global information of the whole MRI image through the incorporation of local-continuous slices. As a result, the model produces high-quality and plausible 3D structural MRIs.
3. The proposed model imputes the missing 3D MRI images directly rather than imputing 2D slices or image derived phenotypes (IDPs). The availability of imputed whole-brain MRI will allow researchers to derive any summary measures of choice using any software of choice without having to adapt or re-run an imputation procedure specific to a certain IDP or software pipeline.
4. The proposed model conditions on a limited set of images (either past or both past and following visits) to generate the missing image for each subject, which is specifically tailored for the analysis of longitudinal data.

These developments collectively contribute to the advancement of longitudinal MRI image imputation and analysis techniques for AD research.

## 2 Methods

### 2.1 Denoising diffusion probabilistic models (DDPM)

DDPM (J. Ho et al., 2020) is a form of latent variable model that approximates the real data distribution${\text{x}}_{0}\;∼p_{data}$with a diffusion process$q({\text{x}}_{t}\;|{\text{x}}_{t-1})$,t∈{1,…,T}, and a denoising process$p_{\theta }({\text{x}}_{t-1}\;|{\text{x}}_{t})$parameterized by weightsθ. The diffusion process transmits$p_{data}$to a standard normal distribution with aT-step Markov chain:

$$q({\text{x}}_{t}\;|{\text{x}}_{t-1})=N\left({\text{x}}_{t};\sqrt{1-\beta_{t}}\;{\text{x}}_{t-1},\;\;\beta_{t}\text{I}\right),$$

where the observed image${\text{x}}_{0}\;\in {\mathbb{R}}^{d}$is assumed to be a draw from$p_{data}$and$\beta_{1},\beta_{2},\cdots ,\beta_{T}$is a fixed variance schedule. The forward sampling at arbitrary time steptis defined as

$$q({\text{x}}_{t}\;|{\text{x}}_{0})=N\left({\text{x}}_{t};\;\sqrt{{\bar{\alpha }}_{t}}{\text{x}}_{0},\;\left(1-{\bar{\alpha }}_{t}\right)\text{I}\right),$$

where$\alpha_{t}=1-\beta_{t}$and${\bar{\alpha }}_{t}=\prod_{s=1}^{t}\alpha_{s}$. Then, a denoising process parameterized by weightsθis leveraged to match the diffusion process at each timesteptwith the following transition kernel:

$$p_{\theta }({\text{x}}_{t-1}\;|{\text{x}}_{t})=N\left({\text{x}}_{t-1};{\tilde{\mu }}_{\theta }\left({\text{x}}_{t},t\right),{\tilde{\beta }}_{t}\text{I}\right),$$

where

$$\begin{array}{l} {\tilde{\mu }}_{\theta }(x_{t},t)=\frac{\sqrt{{\bar{\alpha }}_{t-1}\beta_{t}}}{1-{\bar{\alpha }}_{t}}{\hat{X}}_{0}+\frac{\sqrt{\alpha_{t}(1-{\bar{\alpha }}_{t-1})}}{1-{\bar{\alpha }}_{t}}|x_{t},\\ \;\;\;\;\;\;\;\;\;\;\;\;\;\;\;\;\;{\tilde{\beta }}_{t}=\frac{1-{\bar{\alpha }}_{t-1}}{1-{\bar{\alpha }}_{t}}\beta_{t}. \end{array}$$

The term${\hat{\text{x}}}_{0}$refers to the estimated${\text{x}}_{0}$at timestept,

$${\hat{\text{x}}}_{0}=\frac{{\text{x}}_{t}-\sqrt{1-{\bar{\alpha }}_{t}}\epsilon_{\theta }\left({\text{x}}_{t},t\right)}{\sqrt{{\bar{\alpha }}_{t}}},$$

and$\epsilon_{\theta }$is a neural network, for example, the UNet (Ronneberger et al., 2015), trained to predict noiseϵat steptgiven${\text{x}}_{t}$. The learning objective of DDPM is to optimize the variance bound of$p_{\theta }({\text{x}}_{0})$which can be simplified as the “noise-prediction” loss as inJ. Ho et al. (2020):

$$ℒ(\theta )=E_{t,{\text{x}}_{0}∼p_{data},\epsilon ∼N(\text{0},\text{I})}\left[{\left\|\epsilon -\epsilon_{\theta }\left(\sqrt{\bar{\alpha }}{\text{x}}_{0}+\sqrt{1-{\bar{\alpha }}_{t}}\epsilon ,t\right)\right\|}^{2}\right]$$

### 2.2 Recovery of missing neuroimaging with diffusion models (ReMiND)

In this section, we formulate the longitudinal MRI imputation problem as a generation task conditioned on one or more adjacent MRI images of the designated missing visit. For a given subjectS∈{1,…,N}, we define the longitudinal record ofSas a set of(Image, Existence)pairs arranged in order of visiting timepoint:

$$Record(\text{S})=\left\{\left({\text{x}}^{\text{S},1},\;e^{\text{S},1}\right),\cdots ,\left({\text{x}}^{\text{S},r},\;e^{\text{S},r}\right),\cdots ,\left({\text{x}}^{\text{S},R},\;e^{\text{S},R}\right)\right\},$$

whereRrefers to the number of records contained inRecord(S)if the data were fully observed, that is,R=|Record (S)|, and${\text{x}}^{\text{S},r}\;\in {\mathbb{R}}^{L\times H\times W}$refers to the 3D structural MRI image at ther-th visit with dimensionL×H×W.$e^{\text{S},r}$is the indicator of existence, that is,$e^{\text{S},r}\;=0$means${\text{x}}^{\text{S},r}$is missing;$e^{\text{S},r}\;=1$means${\text{x}}^{\text{S},r}$exists in the observed data. Without loss of generality, we assume ther-th image is missing, that is,$e^{\text{S},r}\;=0$, and its adjacent visits exist, that is,$e^{\text{S},r-1}=e^{\text{S},r+1}=1$. We impute${\text{x}}^{\text{S},r}$by taking its adjacent neighbors as conditions:

$${\text{C}}_{p}\;={\text{x}}^{\text{S},r-1},$$

$${\text{C}}_{p,f}\;=concat\left({\text{x}}^{\text{S},r-1},{\text{x}}^{\text{S},r+1}\right)$$,

where${\text{C}}_{p}$refers to the condition over the past visit and${\text{C}}_{p,f}$refers to the condition over both the past visit and the following visit.concat(⋅,⋅)is the concatenate operation.

Since we aim to recover missing images from neighboring visits where imaging is available, we formulate the longitudinal imputation method as a conditional image generation task. Specifically, ReMiND aims to approximate the distribution of a missing image,${\text{x}}_{0}^{\text{S},r}$, with a parameterized conditional distribution,$p_{\theta }\left({\text{x}}_{0}^{\text{S},r}\;|\text{C}\right)$, whereCis taken to be either${\text{C}}_{p}$or${\text{C}}_{p,f}$. In the form of diffusion and denoising transitions, ReMiND pairs$q\left({\text{x}}_{t-1}^{\text{S},r}|{\text{x}}_{t}^{\text{S},r},{\text{x}}_{0}^{\text{S},r}\right)$with$p_{\theta }\left({\text{x}}_{t-1}^{\text{S},r}|{\text{x}}_{t}^{\text{S},r},\text{C}\right)$at each timestept, where${\text{x}}_{0}^{\text{S},r}\;={\text{x}}^{\text{S},r}$.

To achieve this, the denoising process is re-written as:

$$p_{\theta }({\text{x}}_{t-1}^{\text{S},r}\;|{\text{x}}_{t}^{\text{S},r},\text{C})=N\left({\text{x}}_{t-1}^{\text{S},r};{\tilde{\mu }}_{\theta }\left({\text{x}}_{t}^{\text{S},r},t,\text{C}\right),\frac{1-{\bar{\alpha }}_{t-1}}{1-{\bar{\alpha }}_{t}}\beta_{t}\text{I}\right),$$

where

$$\begin{array}{l} {\tilde{\mu }}_{\theta }\left({\text{x}}_{t}^{\text{S},r},t,\text{C}\right)=\frac{\sqrt{{\bar{\alpha }}_{t-1}}\beta_{t}}{1-{\bar{\alpha }}_{t}}\cdot \frac{{\text{x}}_{t}^{\text{S},r}-\sqrt{1-{\bar{\alpha }}_{t}}\epsilon_{\theta }\left({\text{x}}_{t}^{\text{S},r},t,\text{C}\right)}{\sqrt{{\bar{\alpha }}_{t}}}\\ \;\;\;\;\;\;\;\;\;\;\;\;\;\;\;\;\;\;\;\;\;\;+\frac{\sqrt{\alpha_{t}}\left(1-{\bar{\alpha }}_{t-1}\right)}{1-{\bar{\alpha }}_{t}}{\text{x}}_{t}^{\text{S},r}. \end{array}$$

The diffusion process of ReMiND is the same as a DDPM except we replace the desired data distribution with$q\left({\text{x}}_{0}^{\text{S},r}\right)$. In this way, the learning objective of ReMiND is:

$$ℒ(\theta )=E_{t,{\text{x}}_{0}^{\text{S},r}\;\;∼q\left({\text{x}}_{0}^{\text{S},r}\;\right),\;\epsilon ∼N\left(\text{0},\text{I}\right)}\;\left[\;{\left\|\epsilon -\epsilon_{\theta }\left(\sqrt{{\bar{\alpha }}_{t}}{\text{x}}_{0}^{\text{S},r}\;+\;\sqrt{1-{\bar{\alpha }}_{t}}\epsilon ,t,\text{C}\right)\right\|}^{2}\right].$$ (1)

Henceforth, we denote the model that only relies on the*past*visit as ReMiND-P, that is,$\text{C}={\text{C}}_{p}$, and the model that relies on both the*past*visit and the*following*visit is denoted by ReMiND-PF, that is,$\text{C}={\text{C}}_{p,f}$.

During training,Eq. (1)requires that${\text{x}}^{\text{S},r}$be recovered from random noise if the conditionCis given during the denoising process at each timestep. Once the ReMiND model has converged overEq. (1),$\epsilon_{\theta }$will capture the spatial-temporal dynamics across the missing image and its longitudinal neighboring images. Therefore, for imputation, as long as the condition is provided as prior information, the denoising process will gradually generate the desired missing image. In this work, we only consider models that condition on the immediate adjacent visits, that is,${\text{C}}_{p}$and${\text{C}}_{p,f}$, since these two conditions represent two commonly encountered missingness patterns in real longitudinal data. Our method can also easily be generalized to impute missing visits that occur within longer visit trajectories, that is, multiple past/following visits as conditions.

Normally, modeling high-resolution 3D structural MRIs requires high-capacity 3D convolutional neural networks (Ji et al., 2012;Kopuklu et al., 2019). However, these models are computationally intensive. For instance, the resolution of MRI used in our application is 256×256×172 voxels, which requires enormous GPU memory for training and is generally computationally infeasible. Furthermore, high-capacity models are known to generalize poorly on small-scale datasets such as those available from AD research studies where the number of study participants and images per participant are limited (Zhai et al., 2022). This challenge prompted us to develop 2D convolutional neural networks. A key obstacle in generating 3D MRIs from these 2D networks lies in the inherent limitation of 2D convolutional operations, which are restricted to processing two-dimensional spatial information. This often results in the generation of locally non-continuous 3D MRIs. To address this limitation, we strategically create our training sets to include continuous clips sampled from each segment. This design enables the 2D convolutional networks to effectively capture information across all three dimensions.

In this paper, we mitigate these issues via a parameter-efficient training paradigm by splitting 3D MRIs into uniform local-continuous units and training 2D convolutional neural networks over these units. Concretely, a given 3D structural MRI with the shape of$\text{x}\in {\mathbb{R}}^{L\times H\times W}$has lengthL, widthW, and heightH. We splitxintoKsegments uniformly along theL-axis. Each segment has a resolution of$\frac{L}{K}\times H\times W$. During model training, we first randomly select one slice from each segment. Then, for each selected slice, we concatenate it withJslices immediately before it andJslices immediately after it as the local-continuous clip, which achievesKclips in total with(2J+1)slices for each clip (when sampling random slice from each segment, we skip the firstJand lastJslices of each segment to skip boundary slices when constructingJ-neighbor slices). We then concatenate these clips together, achieving a unit with the shape ofK(2J+1)×H×W. This unit is the local-continuous unit, incorporating both local information (i.e.,2Jlocal slices) and global information (i.e., slices fromKsegments). For each model training step, one of the units is sampled for each MRI and feed into the model. During generation, instead of randomly sampling slices from segmentations, we sample slices in order, forming$\frac{L}{K\left(2J+1\right)}$units for each MRI. Then, we feed these 3D units into the model and collect generations regarding each unit. Finally, we reassemble the generated units back to a 3D MRI with the original shape, i.e.,L×H×W.

## 3 Experiments

### 3.1 Dataset and preprocessing

To illustrate the utility of ReMiND, we leverage longitudinal MRI data that are publicly available from the Alzheimer’s Disease Neuroimaging Initiative (ADNI) (Jack Jr. et al., 2008), which was initiated in 2003 with the goal of facilitating the study of AD. In brief, ADNI enrolled participants between the ages of 55 and 90 who were recruited at 57 sites in the United States and Canada. Informed consent was obtained from all ADNI participants, as approved by the institutional review board at each participating center. The dataset we use comprises T1-weighted MRI with an MP-RAGE sequence at 1.5T from participants who provided data to ADNI 1 on at least three separate visits between September 2005 and May 2017, with a fixed interval of 6 months between each visit. The sample sizes of each clinical diagnosis that we used for training, validation, and testing are presented inTable 1.

**Table 1.** Distribution of observations used for ReMiND-P and ReMiND-PF models overall and stratified by clinical status, including cognitively normal (CN), mild cognitive impairment (MCI), and Alzheimer’s disease (AD).

	ReMiND-P			ReMiND-PF		
Status	Training	Validation	Testing	Training	Validation	Testing
CN	371 (31%)	47 (31%)	42 (28%)	237 (31%)	31 (32%)	29 (26%)
MCI	650 (54%)	78 (52%)	88 (59%)	435 (57%)	54 (55%)	67 (61%)
AD	188 (16%)	26 (17%)	19 (13%)	95 (12%)	13 (13%)	14 (13%)
ALL	1209	151	149	767	98	110

Each value is the sample size followed by the percentage of total observations in the corresponding column. The values in each diagnostic category represent the number of available observations in one of the 10 cross-validation iterations.

We pre-processed the T1-weighted images by following the Advanced Normalization Tools (ANTs) pipeline (Tustison et al., 2019) which includes N4 bias correction as an initial step. For each subject, we first built a single-subject template (SST) using all longitudinal images belonging to that subject followed by rigid registration of each image into the SST space. Next, we rigidly registered each SST to a global template and aligned all within-subject images to the global template by applying the warps from the corresponding SST registration. To reduce computational costs, we rescaled each sagittal slice from 256×256 to 128×128 voxels, resulting in 170×128×128 resolution for each image. Finally, we applied min-max normalization to each image. Since the background voxels in each image have a value of 0, the normalization procedure was considered to be applied exclusively to the voxels within the skull/brain region.

### 3.2 Experiment setting

We used T1-weighted MRI from 632 ADNI 1 participants with clinical status classified as: cognitively normal (CN), mild cognitive impairment (MCI), or AD. We used a 10-fold iterative process for both model selection and evaluation. We randomly partitioned the data into 10 folds. For each iteration, we designated one fold as the test set, while the remaining nine folds were split into training and validation sets in an 8:1 ratio. In each iteration, the training set was used for model fitting, the validation set was used to select values for hyperparameters, and the test set was used to evaluate the model’s performance under the optimal set of hyperparameters identified by the validation set. We trained separate imputation models for two settings: (1) the ReMiND-P model imputes a missing image given the most recent past visit, and (2) the ReMiND-PF model imputes a missing image given the most recent past visit and the future visit that follows the missing time point. We simulated missingness in the dataset for this study by manually selecting some visits from the complete data. Specifically, for the ReMiND-P model, every second visit was considered as a missing data point. On the other hand, for the ReMiND-PF model, the middle visit was regarded as missing data among every three observed timepoints. We refer to the selected missing image, which actually exists in the dataset, as the “observed image” in this study.

To assess the performance of the ReMiND models, we first calculated the structural similarity index (SSIM) and the peak signal-to-noise ratio (PSNR) to quantify the proximity of the imputed images to the observed images. SSIM is an algorithm that checks the similarity between two images based on three factors: luminance, contrast, and structure. It is designed to better suit the human visual system and capture perceptual changes in the image. SSIM ranges from -1 to 1, where 1 means perfect similarity (Wang et al., 2018). PSNR is a ratio that measures the amount of noise or distortion introduced by compression or reconstruction. It is based on the mean squared error between the two images. The higher the PSNR, the better the quality of the image (Hore & Ziou, 2010). We further compared regional brain volumes estimated using two common pipelines: (1) ANTs (Tustison et al., 2019) and (2) FreeSurfer (Fischl, 2012). We employed the error rate and progression rate as metrics to facilitate the comparison. The error rate, calculated as$\left|{\hat{y}}_{i}-y_{i}\right|/y_{i}$, compares${\hat{y}}_{i}$, the estimated volume of a specific region from the imputed image, to that from the observed image,$y_{i}$. A lower error rate indicates better performance. The progression rate, on the other hand, measures the rate of volume decline, reflecting brain atrophy between adjacent visits. For imputed images, the progression rate is computed as$\left|{\hat{y}}_{i}-y_{i-1}\right|/y_{i-1}$. Here,$y_{i-1}$represents the volume of the region at the previous visit obtained from the observed image, and$\left|{\hat{y}}_{i}-y_{i-1}\right|$represents the change in brain volume obtained by contrasting the estimated volume from the imputed image at visitito the volume from the observed image at visiti−1. Similarly, the gold-standard progression rate is calculated from observed images as$\left|y_{i}-y_{i-1}\right|/y_{i-1}$. The smaller the difference between the progression rate using an observed image and the progression rate using an imputed image signifies better performance.

Furthermore, we studied the relative performance of ReMiND versus two comparator models: naive imputation by forward filling (Naive) and imputation using an autoencoder (AE). The Naive-P model simply predicted all missing images to be the same as the past observed images. The Naive-PF model predicted the missing images by averaging the adjacent past and future images. AE models are widely used for image processing and generation (Baur et al., 2021;Myronenko, 2019;Xia et al., 2021;Zhao et al., 2017). We trained AE models to take as input the past or past and following visits and then minimize the$\ell_{2}$loss between the output (i.e., imputed image) and the target “missing” MRI. Thus, AE-P refers to taking the past visit as input and AE-PF refers to taking both the past visit and the following visit as input to impute the missing MRI. We additionally compared performance of each imputation method and processing pipeline separately by clinical diagnosis group. However, training utilized data pooled from all groups.

### 3.3 Implementation details

We adopted a modified UNet (Dhariwal & Nichol, 2021) with larger capacity and additional attention blocks. Models were trained in 200,000 steps with Adam (Kingma & Ba, 2015) as the optimizer. We followed the UNet architecture described inJ. Ho et al. (2020)except for the model size, where we adopted the channel multiplier as 64 for ReMiND-P and 128 for ReMiND-PF since ReMiND-PF consists of larger conditions. We trained both ReMiND-P and ReMiND-PF in 200,000 steps with the Adam optimizer (Kingma & Ba, 2015). The learning rate was set to 0.0001, and the batch size was set to 16 during the training. We adopted the same U-Net architecture as ReMiND for the AE models. Since the AE models tend to converge easily, we trained the AEs until the loss stopped decreasing (around 20,000 steps) with the learning rate set as 1e-4. All experiments were conducted on servers consisting of one Nvidia RTX 3090 GPU, one Intel i9-12900F CPU, and 32G RAM. PyTorch (Paszke et al., 2019) was used as the deep learning framework in our implementation.

## 4 Results

### 4.1 Whole-brain imputed images

To qualitatively evaluate the ReMiND-generated images, we visually compared the imputed images with their corresponding observed images for one subject inFigure 2. The presented images were generated with the models conditioned on both past and following images, as ReMiND-PF outperformed the ReMiND-P model on the quantitative measures which we will report next. To visually demonstrate the superior performance of the ReMiND-PF method, we display corresponding slices from the images generated using the Naive-PF and AE-PF models. For all methods, images were intentionally generated with the skull on to prioritize flexibility in downstream analyses. That is, researchers working with the imputed image could apply their brain extraction method of choice.

**Fig. 2.**
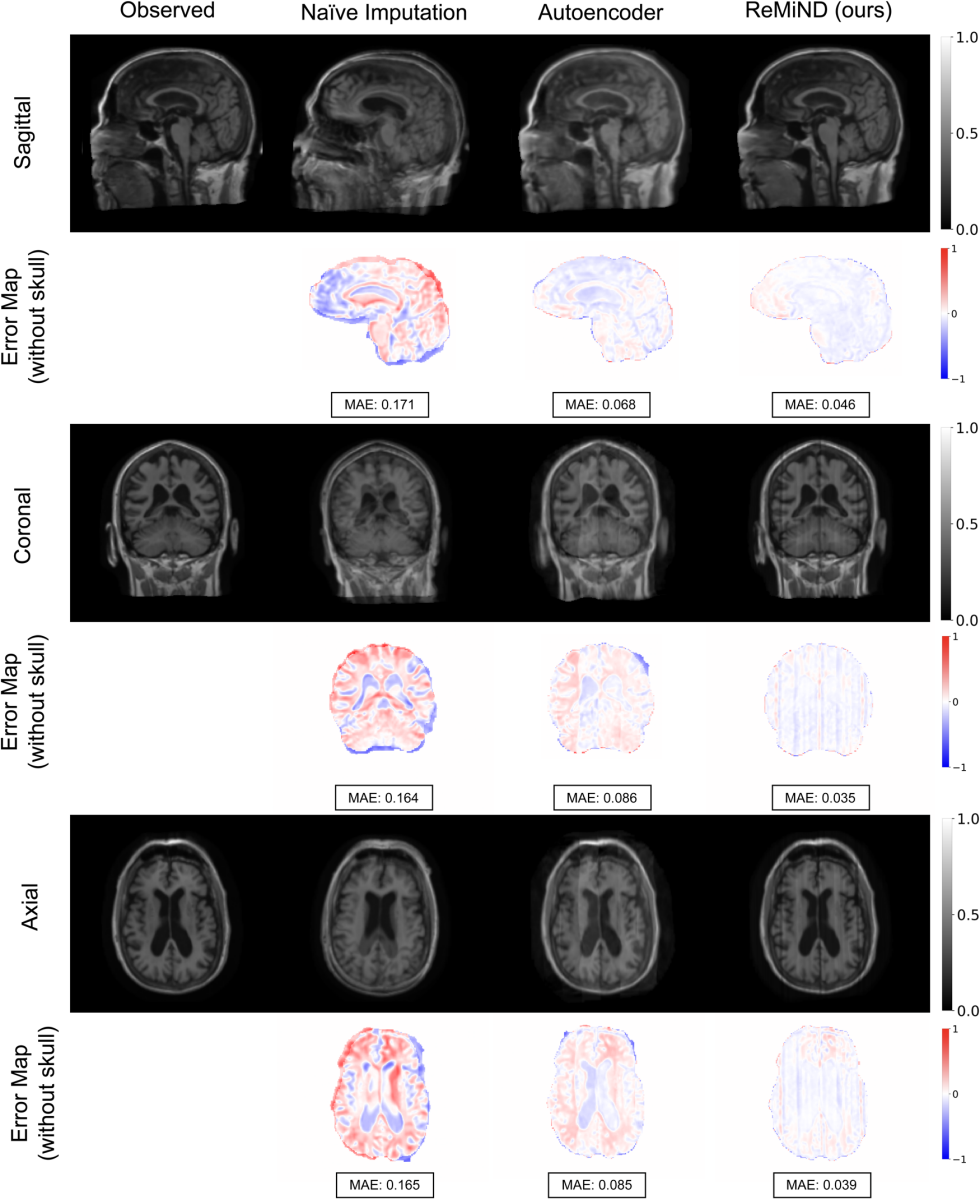
Qualitative comparison of observed images and imputed T1-weighted MRI which were generated by Naive-PF, AE-PF, and the proposed ReMiND-PF models. The first, third, and fifth rows show the generated images from sagittal, coronal, and axial views, respectively. The second, fourth, and last row shows the error maps of brain voxelwise differences between the observed and generated images. MAE indicates the mean absolute error.

As shown inFigure 2, images generated with the ReMiND model are visually more similar to the observed images compared to the other two imputation methods. The Naive-PF imputed images have several blurry areas and imprecise skulls due to averaging across rigidly registered images from different visits. Images imputed using the autoencoder model are marginally better than those generated using the naive approach but still exhibit undesirable artifacts and fuzzy edges. Compared to the other methods, the ReMiND model generated images with sharper edges and finer anatomical details in critical gray matter regions for this individual. Based on the qualitative comparison inFigure 2, our method appears to capture important anatomical structures such as the cortical gray matter with high integrity. The differing performance of the three imputation methods is further highlighted by the error images of brain voxel-wise differences between the generated and observed images. The Naive imputation exhibited the largest dissimilarity between observed and imputed images, while ReMiND preserved fine structural details resulting in small voxelwise differences across the brain.

Table 2quantifies the proximity of the imputed images to the observed images at the target visit, considering images with skull voxels excluded. Across all clinical statuses, ReMiND models outperformed the Naive and AE models with respect to SSIM and PSNR values. This finding held both when imputing conditional on the past image and conditional on the past and following images. The ReMiND-PF model had both larger SSIM and PSNR than the ReMiND-P model, suggesting the model performs better when more than one time point is available to condition on for imputation. In addition to testing the models on the whole testing dataset, we evaluated the performance of all methods separately among groups of subjects categorized as CN, MCI, and AD. We found only minor differences between the all-status group and each clinical status group for both SSIM and PSNR. Our findings suggest that the methods considered can effectively impute missing structural MRI data for patients across the spectrum of clinical severity.

**Table 2.** Comparison of model performance averaged across 10 test sets.

Model	Images	CN	MCI	AD	ALL
SSIM (higher = better)					
Naive-P	Skull removed	0.899 ± 0.015	0.899 ± 0.005	0.914 ± 0.007	0.901 ± 0.005
AE-P	Skull removed	0.913 ± 0.008	0.912 ± 0.006	0.911 ± 0.002	0.912 ± 0.006
ReMiND-P	Skull removed	**0.974** ± **0.003**	**0.974** ± **0.001**	**0.974** ± **0.002**	**0.974** ± **0.001**
Naive-PF	Skull removed	0.894 ± 0.025	0.886 ± 0.013	0.893 ± 0.008	0.890 ± 0.006
AE-PF	Skull removed	0.919 ± 0.006	0.914 ± 0.0002	0.916 ± 0.005	0.916 ± 0.002
ReMiND-PF	Skull removed	**0.984** ± **0.002**	**0.982** ± **0.0004**	**0.983** ± **0.0009**	**0.983** ± **0.0002**
PSNR (dB) (higher = better)					
Naive-P	Skull removed	22.868 ± 0.754	24.644 ± 0.713	23.217 ± 0.632	23.217 ± 0.632
AE-P	Skull removed	25.911 ± 0.768	26.820 ± 0.314	26.962 ± 0.660	26.962 ± 0.660
ReMiND-P	Skull removed	**29.078** ± **0.781**	**28.908** ± **0.458**	**28.824** ± **0.651**	**28.960** ± **0.434**
Naive-PF	Skull removed	23.743 ± 2.957	22.537 ± 1.353	23.457 ± 1.026	23.097 ± 0.571
AE-PF	Skull removed	27.040 ± 0.590	26.613 ± 0.100	27.954 ± 0.662	27.792 ± 0.232
ReMiND-PF	Skull removed	**31.786** ± **0.399**	**31.175** ± **0.218**	**31.857** ± **0.343**	**31.449** ± **0.090**

Performance was measured with structural similarity index (SSIM) and peak signal-to-noise ratio (PSNR) in decibels (dB) on the generated MRI images with the skull removed (i.e., voxels within the group template brain mask only).

Performance was evaluated overall and separately by clinical group (CN, MCI, and AD). P indicates the imputation method conditioned on the most recent past image. PF indicates the imputation method conditioned on both the most recent past image and the closest following image. AE indicates imputation using an autoencoder. Bold values indicate the best performing imputation method for a given clinical diagnosis group within the P or PF condition.

The results for images with the skull are provided inSupplementary Table 1. SSIM and PSNR values were lower when computed on brain images with the skull included compared to images without skull voxels. This is likely due to across-subject heterogeneity in extra-cerebral voxels that display the neck and facial features. Since extra-cerebral regions are not important for studying the effects of AD in the brain over time, evaluation of ReMiND and other methods should focus on metrics with the skull removed.

### 4.2 Evaluation of volumetric features extracted from generated images

We evaluated the performance of the proposed ReMiND models with respect to volumetric features extracted using the ANTs pipeline (Tustison et al., 2014) and FreeSurfer’s pipeline (Fischl, 2012). The version of ANTs is 0.3.4 for ANTsPy. The version of FS is 7.3.2. The segmentation atlas of ANTs is MNI-ICBM 152 nonlinear 2009 and the segmentation atlas of FS is Freesurfer Automatic Segmentation (Aseg). We primarily focused on 28 regions defined by the MNI atlas (Fonov et al., 2011) that have been shown to be associated with AD pathology in the brain. We also choose the 28 regions from the Aseg atlas for FS whose names are identical to those of 28 MNI regions. Detailed region names are provided inSupplementary Table 2. Results inTable 3are based on the average across these 28 regions and are also averaged across the 10 testing sets.

**Table 3.** Comparison of the test performance of imputation methods with respect to volumetric features extracted from whole-brain imputed images.

Models	Error rate (ANTs)	Error rate (FreeSurfer)	Progression rate (ANTs)	Progression rate (FreeSurfer)
Naive-P	0.0310 ± 0.0028	0.1877 ± 0.0033	0	0
			(–)	(–)
AE-P	0.0284 ± 0.0002	0.1078 ± 0.0004	0.0422 ± 0.0038	0.1358 ± 0.0030
			(-0.0121)	(-0.1328)
ReMiND-P	0.0228 ± 0.0003	0.0892 ± 0.0029	0.0361 ± 0.0033	0.1024 ± 0.0004
			(-0.006)	(-0.051)
Naïve-PF	0.0218 ± 0.0009	0.1393 ± 0.0032	0.0365 ± 0.0012	0.1842 ± 0.0031
			(-0.0157)	(-0.1332)
AE-PF	0.0192 ± 0.0008	0.0937 ± 0.0019	0.0231 ± 0.0009	0.0949 ± 0.0013
			(-0.0023)	(-0.0439)
ReMiND-PF	**0.0178** ± **0.0002**	**0.0509** ± **0.0003**	**0.0226** ± **0.0006**	**0.0872** ± **0.0018**
			(-0.0017)	(-0.0362)

Results were averaged across the 10 test sets and averaged across all regions defined by the MNI atlas for ANTs and Aseg atlas for FreeSurfer pipeples. Lower error rate indicates better performance. The values in () are differences between the observed progression rate (using observed images at both time points) and the estimated progression rate using the imputed image at the latter time point. Lower difference means better performance. P indicates past image. PF indicates past and following images. The best test results across all methods and models are bolded.

We found the error rates were lower for PF models compared to P models across all three methods (Naive, AE, and ReMiND). These results suggest models that condition on past and following images perform better at the task of generating accurate missing MRI images at the designated visits, which is likely explained by the additional information of the subsequent observed image. Under each experiment setting (P and PF), ReMiND models had the lowest error rates compared with the two comparator methods, and the Naive method had the highest error rates. Furthermore, ANTs-based error rates were lower across all methods and experimental settings compared to FreeSurfer as expected based on previous work (Tustison et al., 2014).

Progression rates for the Naive-P model were all zero (Table 3) because the Naive-P model generates the missing image simply by carrying forward the past image. The ReMiND-P model had lower differences in observed and imputed progression than AE-P using both the ANTs and FreeSurfer pipelines. Furthermore, ReMiND-PF models exhibited the lowest differences between the observed and imputed progression rates for both volume estimation pipelines. FreeSurfer-based differences in progression rates were larger than ANTs-based differences. We also conducted a comparison of the average progression rates within the CN, MCI, and AD groups separately to assess the effectiveness of our imputation method (Supplementary Table 3). A greater difference in average progression rates between these clinical groups would suggest enhanced imputation effectiveness. Our results demonstrate that the ReMiND models exhibit the greatest magnitude of differences across clinical sub-groups, thereby indicating their superior performance over alternative approaches.

**Fig. 3.**
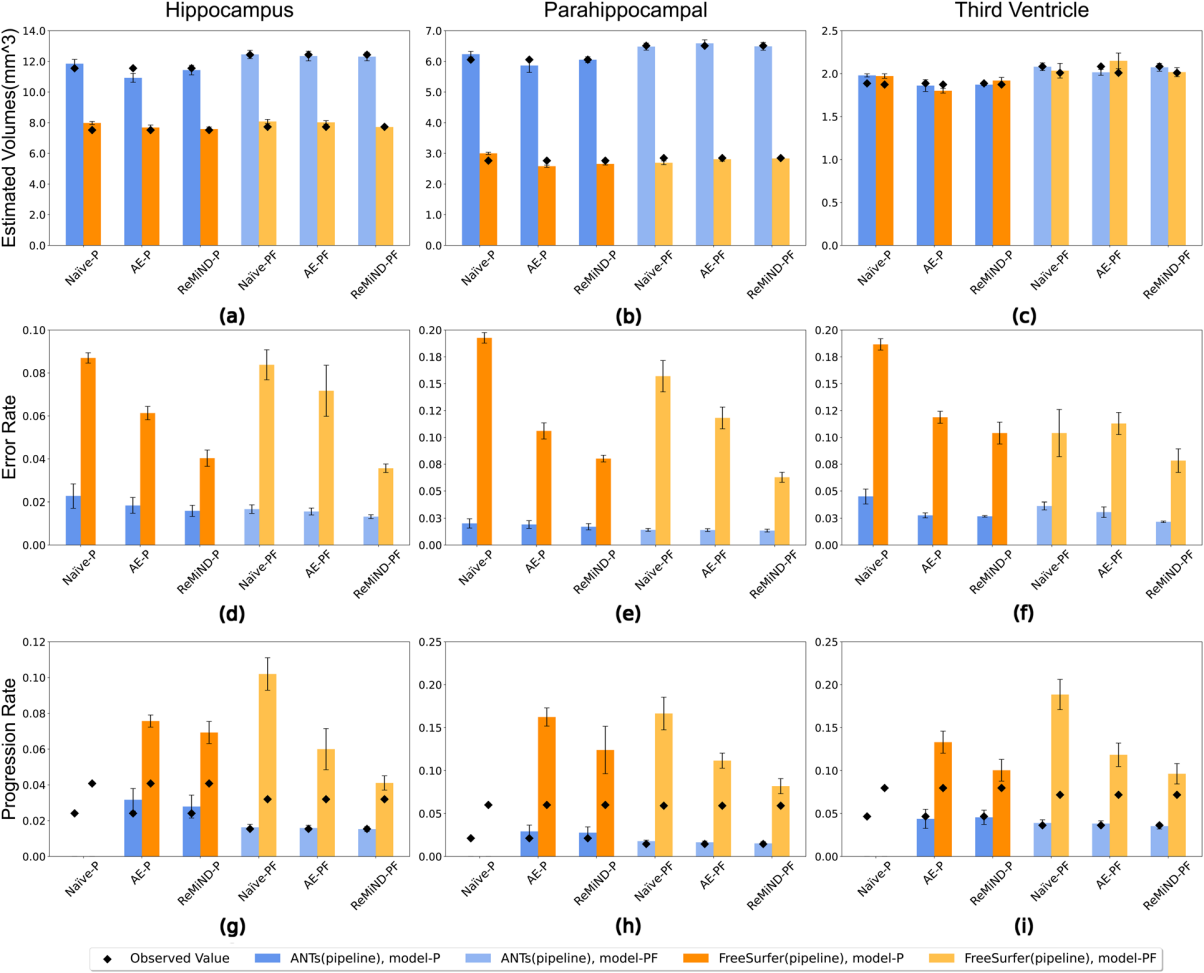
Comparison of volumetric features for three brain regions estimated on observed MRI images and MRI images generated from naive imputation, autoencoder, and ReMiND models. Results were averaged across 10 test sets. Error bars show standard derivation across test sets. Panels (a-c) display average volumes estimated from images generated with different models, with the black diamonds representing the average volume of each region obtained from observed images. Panels (d-f) present the comparison of error rates, where a lower value indicates better performance. In panels (g-i), the progression rate calculated using imputed images is displayed, with the black diamonds representing the progression rate calculated using observed images. The volumes were estimated and compared using the ANTs and FreeSurfer pipelines. P indicates the past image model. PF indicates models that used past and following images.

In addition to results averaged across all 28 prioritized brain regions, we report results from the hippocampus, parahippocampal region, and the third ventricle individually inFigure 3. The figure displays total estimated volume (in mm^3^), error rate, and progression rate for all imputation methods and both P and PF models. All results were averaged across 10 test sets. Not surprisingly, the performance reflects the results inTable 3. ReMiND models outperform both comparator imputation approaches. Specifically,Figure 3a-cshows that ReMiND models exhibit smaller discrepancies between the estimated and observed values, which are indicated as black diamonds in the figure, compared to Naive and AE models.Figure 3d-fdemonstrates that images generated by ReMiND models have lower error rates than those produced by both the Naive and AE imputation methods across the two pipelines. The third row ofFigure 3g-idemonstrates that the ReMiND-P and ReMiND-PF models produce the smallest differences between imputed and observed progression rates across imputation methods. Although the estimated volumes may not exhibit significant visual distinctions, the observed differences in error rate and progression rates in the plot are primarily influenced by the difference between the estimated and observed values for each method.

To further evaluate the performance of the ReMiND models for imputing missing images and carrying out downstream tasks, we estimated 1-year atrophy rates in individuals clinically diagnosed with AD. This assessment specifically focused on three brain regions: the hippocampus, the parahippocampal region, and the third ventricle. We conducted comparisons under three scenarios: (1) all subjects had complete data for their baseline, 6-month, and 12-month visits; (2) for a random half of the subjects, data at the 12-month visit were intentionally omitted. We estimated their atrophy rates based on the 6-month data and then doubled these estimates to approximate their atrophy rates at 12 months (i.e., linear prediction); (3) using the ReMiND model to impute images for those missing their 12-month data. We then calculated 1-year atrophy rates under each scenario. As illustrated inSupplementary Table 4, atrophy rates derived using ReMiND more closely align with results based on complete 12-month data compared to scenario 2. This demonstrates that the imputation approach has the potential to enhance downstream analyses, such as the estimation of atrophy rates in Alzheimer’s disease.

## 5 Conclusions

In this study, we introduced an innovative diffusion model-based framework for 3D longitudinal structural MRI imputation with an aim to generate missing 3D brain images at a specific single visit. To achieve this, the 3D image is partitioned into uniform local-continuous clips, with each clip consisting of three consecutive slices of the MRI image. Notably, our method distinguishes itself from conventional approaches by employing sets of 3D clips as input, thereby enhancing the proposed model’s ability to capture comprehensive global information from the entire MRI dataset during the training phase. The model utilizes single past or both past and following visits in the temporal direction to impute missing structural MRIs. Experimental results showed that our model consistently outperformed two comparator techniques: last image carried forward (i.e., forward filling) and imputation using an autoencoder. The comparison of similarity metrics highlighted our proposed model’s ability to accurately generate high-quality images for imputing missingness. We compared the volumes of a set of image derived phenotypes (IDPs) estimated from the generated and observed images using two different pipelines (ANTs and FreeSurfer). The relatively low error rates and accurately estimated rates of change in volume over time demonstrated that our proposed models can generate plausible whole-brain, 3D structural MRI data.

Importantly, by imputing full 3D images rather than IDPs directly, researchers can flexibly utilize the imputed images in downstream statistical or predictive models, including using the generated images for further imputation of missing data in the temporal dimension. The proposed models may be beneficial for generating missing data in various other medical imaging contexts. Moreover, high-quality MRI images can provide better visualization of brain structures enabling earlier and more precise detection of pathological changes associated with the disease. Subtle changes in brain structures, such as the hippocampus, can be indicative of early AD. With the generative model trained on a large dataset, imputing longitudinal imaging data when participants miss a scheduled visit may provide an early indicator of subsequent changes in the brain. In future studies, such predictions of brain atrophy may be useful for encouraging patients to remain engaged in the study so they can compare their actual brain changes with changes predicted by the model. Furthermore, consistent and high-quality imaging will be crucial for evaluating the effect of new treatments. For example, longitudinal imaging can be used to monitor whether a new drug slows, halts, or even reverses brain atrophy.

Across all experiments, the ReMiND-PF model, which conditioned on both past and future visits, outperformed the ReMiND-P model which solely relied on the past timepoint. This may be due to the ReMiND-PF model’s use of more information to generate images, highlighting the importance of quantity and quality of available information for imputing missingness. We evaluated the performance of the models on each clinical status group (CN, MCI, AD) and a combined all-status group and found that both ReMiND-P and ReMiND-PF performed well in all scenarios.

One limitation of our study is that we did not perform skull-stripping prior to training the diffusion models. Although the ReMiND model shows improved performance when skull voxels are excluded following image generation with the skull, our study does not provide direct empirical evidence to support the claim that skull-stripping prior to model training could enhance the accuracy of predictions. Therefore, additional studies are needed to robustly demonstrate that diffusion models can accurately handle the presence of rigid structures such as skull voxels without compromising performance. Another limitation of our study lies in the utilization of either a single past image or both past and following images as conditional information for missingness imputation while ensuring a 6-month interval between consecutive visits. Future investigations could involve expanding the approach to incorporate multiple images with diverse visit interval timing. In our longitudinal study, models are configured to operate with a fixed 6-month time interval between images. Future work could focus on extending these models to accommodate varying time intervals. Our study was conducted exclusively on single-scanner 1.5T images, and we have not evaluated the effectiveness of the imputation method on 3T images. Therefore, the performance of our imputation method on higher field strength images should be investigated in future studies. Additionally, there is potential for further research on downstream analyses, such as using the imputed imaging trajectories to develop models that predict the progression of Alzheimer’s disease.

## Supplementary Material

Supplementary Material

## Data Availability

Data used in this study are available through the public dataset (https://adni.loni.usc.edu/). The code used in this paper can be found athttps://github.com/jinhaoduan/ReMiND.

## References

[b1] Arnold , L. , Rebecchi , S. , Chevallier , S. , & Paugam-Moisy , H. ( 2011 ). An introduction to deep learning . In European symposium on artificial neural networks (ESANN) . https://hal.science/hal-01352061/

[b2] Aviles-Rivero , A. I. , Runkel , C. , Papadakis , N. , Kourtzi , Z. , & Schönlieb , C.-B. ( 2022 ). Multi-modal hypergraph diffusion network with dual prior for Alzheimer classification . In Medical image computing and computer assisted intervention–MICCAI 2022: 25th international conference, Singapore, September 18–22, 2022, Proceedings, Part III (pp. 717 – 727 ). 10.1007/978-3-031-16437-8_69

[b3] Baur , C. , Denner , S. , Wiestler , B. , Navab , N. , & Albarqouni , S. ( 2021 ). Autoencoders for unsupervised anomaly segmentation in brain mr images: A comparative study . Medical Image Analysis , 69 , 101952 . 10.1016/j.media.2020.101952 33454602

[b4] Campos , S. , Pizarro , L. , Valle , C. , Gray , K. R. , Rueckert , D. , & Allende , H. ( 2015 ). Evaluating imputation techniques for missing data in ADNI: A patient classification study . In Progress in pattern recognition, image analysis, computer vision, and applications: 20th Iberoamerican congress, CIARP 2015, Montevideo, Uruguay, November 9–12, 2015, Proceedings 20 (pp. 3 – 10 ). 10.1007/978-3-319-25751-8_1

[b5] De Strooper , B. , & Karran , E. ( 2016 ). The cellular phase of Alzheimer’s disease . Cell , 164 ( 4 ), 603 – 615 . 10.1016/j.cell.2015.12.056 26871627

[b6] Dhariwal , P. , & Nichol , A. ( 2021 ). Diffusion models beat GANs on image synthesis . Advances in Neural Information Processing Systems , 34 , 8780 – 8794 . 10.48550/arXiv.2105.05233

[b7] Fischl , B. ( 2012 ). Freesurfer . Neuroimage , 62 ( 2 ), 774 – 781 . 10.1016/j.neuroimage.2012.01.021 22248573 PMC3685476

[b8] Fonov , V. , Evans , A. C. , Botteron , K. , Almli , C. R. , McKinstry , R. C. , Collins , D. L. , & Brain Development Cooperative Group . ( 2011 ). Unbiased average age-appropriate atlases for pediatric studies . Neuroimage , 54 ( 1 ), 313 – 327 . 10.1016/j.neuroimage.2010.07.033 20656036 PMC2962759

[b9] Goodfellow , I. , Pouget-Abadie , J. , Mirza , M. , Xu , B. , Warde-Farley , D. , Ozair , S. , Courville , A. , & Bengio , Y. ( 2020 ). Generative adversarial networks . Communications of the ACM , 63 ( 11 ), 139 – 144 . 10.1145/3422622

[b10] Hardy , S. E. , Allore , H. , & Studenski , S. A. ( 2009 ). Missing data: A special challenge in aging research . Journal of the American Geriatrics Society , 57 ( 4 ), 722 – 729 . 10.1111/j.1532-5415.2008.02168.x 19220562 PMC2695652

[b12] Ho , J. , Jain , A. , & Abbeel , P. ( 2020 ). Denoising diffusion probabilistic models . Advances in Neural Information Processing Systems , 33 , 6840 – 6851 . 10.48550/arXiv.2006.11239

[b13] Ho , J. , Saharia , C. , Chan , W. , Fleet , D. J. , Norouzi , M. , & Salimans , T. ( 2022 ). Cascaded diffusion models for high fidelity image generation . Journal of Machine Learning Research , 23 ( 47 ), 1 – 33 . https://www.jmlr.org/papers/volume23/21-0635/21-0635.pdf

[b14] Ho , N.-H. , Yang , H.-J. , Kim , J. , Dao , D.-P. , Park , H.-R. , & Pant , S. ( 2022 ). Predicting progression of Alzheimer’s disease using forward-to-backward bi-directional network with integrative imputation . Neural Networks , 150 , 422 – 439 . 10.1016/j.neunet.2022.03.016 35364417

[b15] Hore , A. , & Ziou , D. ( 2010 ). Image quality metrics: PSNR vs. SSIM . In 2010 20th international conference on pattern recognition (pp. 2366 – 2369 ). 10.1109/icpr.2010.579

[b16] Ibrahim , J. G. , Chu , H. , & Chen , M.-H. ( 2012 ). Missing data in clinical studies: Issues and methods . Journal of Clinical Oncology , 30 ( 26 ), 3297 . 10.1200/jco.2011.38.7589 22649133 PMC3948388

[b17] Isola , P. , Zhu , J.-Y. , Zhou , T. , & Efros , A. A. ( 2017 ). Image-to-image translation with conditional adversarial networks . In Proceedings of the IEEE conference on computer vision and pattern recognition (pp. 5967 – 5976 ). 10.1109/cvpr.2017.632

[b18] Jack Jr. , C. R. , Bernstein , M. A. , Fox , N. C. , Thompson , P. , Alexander , G. , Harvey , D. , Borowski , B. , Britson , P. J. , Whitwell L. , J., Ward , C. , Dale , A. M. , Felmlee , J. P. , Gunter , J. L. , Hill , D. L. , Killiany , R. , Schuff , N. , Fox-Bosetti , S. , Lin , C. , Studholme , C. , … Weiner , M. W. ( 2008 ). The Alzheimer’s disease neuroimaging initiative (ADNI): MRI methods . Journal of Magnetic Resonance Imaging , 27 ( 4 ), 685 – 691 . 10.1002/jmri.21049 18302232 PMC2544629

[b19] Ji , S. , Xu , W. , Yang , M. , & Yu , K. ( 2012 ). 3D convolutional neural networks for human action recognition . IEEE Transactions on Pattern Analysis and Machine Intelligence , 35 ( 1 ), 221 – 231 . 10.1109/tpami.2012.59 22392705

[b20] Junninen , H. , Niska , H. , Tuppurainen , K. , Ruuskanen , J. , & Kolehmainen , M. ( 2004 ). Methods for imputation of missing values in air quality data sets . Atmospheric Environment , 38 ( 18 ), 2895 – 2907 . 10.1016/j.atmosenv.2004.02.026

[b21] Kingma , D. P. , & Ba , J. ( 2015 ). Adam: A method for stochastic optimization . In International conference on learning representations (ICLR) . 10.48550/arXiv.1412.6980

[b22] Kingma , D. P. , & Welling , M. ( 2013 ). Auto-encoding variational bayes . arXiv preprint arXiv:1312.6114 . 10.48550/arXiv.1312.6114

[b23] Kong , Z. , Ping , W. , Huang , J. , Zhao , K. , & Catanzaro , B. ( 2021 ). Diffwave: A versatile diffusion model for audio synthesis . In International conference on learning representations . https://openreview.net/forum?id=a-xFK8Ymz5J

[b24] Kopuklu , O. , Kose , N. , Gunduz , A. , & Rigoll , G. ( 2019 ). Resource efficient 3D convolutional neural networks . In Proceedings of the IEEE/CVF international conference on computer vision workshops (pp. 1910 – 1919 ). 10.1109/iccvw.2019.00240

[b25] Lipton , Z. C. , Kale , D. C. , & Wetzel , R. ( 2016 ). Modeling missing data in clinical time series with RNNs . Proceedings of Machine Learning for Healthcare , 56 , 253 – 270 . https://proceedings.mlr.press/v56/Lipton16.pdf

[b26] Lo , R. Y. , & Jagust , W. J. ( 2012 ). Predicting missing biomarker data in a longitudinal study of Alzheimer disease . Neurology , 78 ( 18 ), 1376 – 1382 . 10.1212/WNL.0b013e318253d5b3 22491869 PMC3345787

[b27] Lugmayr , A. , Danelljan , M. , Romero , A. , Yu , F. , Timofte , R. , & Van Gool , L. ( 2022 ). Repaint: Inpainting using denoising diffusion probabilistic models . In Proceedings of the IEEE/CVF conference on computer vision and pattern recognition (pp. 11451 – 11461 ). 10.1109/cvpr52688.2022.01117

[b28] Myronenko , A. ( 2019 ). 3D MRI brain tumor segmentation using autoencoder regularization . In Brainlesion: Glioma, multiple sclerosis, stroke and traumatic brain injuries: 4th international workshop, BrainLes 2018, held in conjunction with MICCAI 2018, Granada, Spain, September 16, 2018, revised selected papers, Part II (pp. 311 – 320 ). 10.1007/978-3-030-11726-9_28

[b29] Nguyen , M. , He , T. , An , L. , Alexander , D. C. , Feng , J. , Yeo , B. T. , & Initiative Alzheimer’s Disease Neuroimaging . ( 2020 ). Predicting Alzheimer’s disease progression using deep recurrent neural networks . NeuroImage , 222 , 117203 . 10.1016/j.neuroimage.2020.117203 32763427 PMC7797176

[b30] Paszke , A. , Gross , S. , Massa , F. , Lerer , A. , Bradbury , J. , Chanan , G. , Killeen , T. , Lin , Z. , Gimelshein , N. , Antiga , L. , Desmaison , A. , Kopf , A. , Yang , E. , DeVito , Z. , Raison , M. , Tejani , A. , Chilamkurthy , S. , Steiner , B. , Fang , L. , … Chintala , S. ( 2019 ). Pytorch: An imperative style, high-performance deep learning library . In Advances in neural information processing systems (pp. 8024 – 8035 ). http://papers.neurips.cc/paper/9015-pytorch-an-imperative-style-high-performance-deep-learning-library.pdf

[b31] Peng , C. , Guo , P. , Zhou , S. K. , Patel , V. M. , & Chellappa , R. ( 2022 ). Towards performant and reliable undersampled mr reconstruction via diffusion model sampling . In Medical image computing and computer assisted intervention–MICCAI 2022: 25th international conference, Singapore, September 18–22, 2022, Proceedings, Part VI (pp. 623 – 633 ). 10.1007/978-3-031-16446-0_59

[b32] Pinaya , W. H. , Graham , M. S. , Gray , R. , Da Costa , P. F. , Tudosiu , P.-D. , Wright , P. , Mah , Y. H. , MacKinnon , A. D. , Teo , J. T. , Jager , R. , Werring , D. , Rees , G. , Nachev , P. , Ourselin , S. , & Cardoso , M. J. ( 2022 ). Fast unsupervised brain anomaly detection and segmentation with diffusion models . In Medical image computing and computer assisted intervention–MICCAI 2022: 25th international conference, Singapore, September 18–22, 2022, Proceedings, Part VIII (pp. 705 – 714 ). 10.1007/978-3-031-16452-1_67

[b33] Ronneberger , O. , Fischer , P. , & Brox , T. ( 2015 ). U-net: Convolutional networks for biomedical image segmentation . In Medical image computing and computer-assisted intervention–MICCAI 2015: 18th international conference, Munich, Germany, October 5–9, 2015, Proceedings, Part III (pp. 234 – 241 ). 10.1007/978-3-319-24574-4_28

[b34] Shamsolmoali , P. , Zareapoor , M. , Granger , E. , Zhou , H. , Wang , R. , Celebi , M. E. , & Yang , J. ( 2021 ). Image synthesis with adversarial networks: A comprehensive survey and case studies . Information Fusion , 72 , 126 – 146 . 10.1016/j.inffus.2021.02.014

[b35] Shishegar , R. , Cox , T. , Rolls , D. , Bourgeat , P. , Doré , V. , Lamb , F. , Robertson , J. , Laws , S. M. , Porter , T. , Fripp , J. , Tosun , D. , Maruff , P. , Savage , G. , Rowe , C. C. , Masters , C. L. , Weiner , M. W. , Villemagne , V. L. , & Burnham , S. C. ( 2021 ). Using imputation to provide harmonized longitudinal measures of cognition across AIBL and ADNI . Scientific Reports , 11 ( 1 ), 23788 . 10.1038/s41598-021-02827-6 34893624 PMC8664816

[b36] Tustison , N. J. , Cook , P. A. , Klein , A. , Song , G. , Das , S. R. , Duda , J. T. , Kandel , B. M. , van Strien , N. , Stone , J. R. , Gee , J. C. , & Avants , B. B. ( 2014 ). Large-scale evaluation of ants and freesurfer cortical thickness measurements . Neuroimage , 99 , 166 – 179 . 10.1016/j.neuroimage.2014.05.044 24879923

[b37] Tustison , N. J. , Holbrook , A. J. , Avants , B. B. , Roberts , J. M. , Cook , P. A. , Reagh , Z. M. , Duda , J. T. , Stone , J. R. , Gillen , D. L. , Yassa , M. A. , & Alzheimer’s Disease Neuroimaging Initiative . ( 2019 ). Longitudinal mapping of cortical thickness measurements: An Alzheimer’s disease neuroimaging initiative-based evaluation study . Journal of Alzheimer’s Disease , 71 ( 1 ), 165 – 183 . 10.3233/jad-190283 PMC10204115 31356207

[b38] van Oostveen , W. M. , & de Lange , E. C. ( 2021 ). Imaging techniques in Alzheimer’s disease: A review of applications in early diagnosis and longitudinal monitoring . International Journal of Molecular Sciences , 22 ( 4 ), 2110 . 10.3390/ijms22042110 33672696 PMC7924338

[b39] Voleti , V. , Jolicoeur-Martineau , A. , & Pal , C. ( 2022 ). Masked conditional video diffusion for prediction, generation, and interpolation . arXiv Preprint arXiv:2205.09853 . 10.48550/arXiv.2205.09853

[b40] Wang , T.-C. , Liu , M.-Y. , Zhu , J.-Y. , Tao , A. , Kautz , J. , & Catanzaro , B. ( 2018 ). High-resolution image synthesis and semantic manipulation with conditional gans . In Proceedings of the IEEE conference on computer vision and pattern recognition (pp. 8798 – 8807 ). 10.1109/cvpr.2018.00917

[b41] Wolleb , J. , Bieder , F. , Sandkühler , R. , & Cattin , P. C. ( 2022 ). Diffusion models for medical anomaly detection . In Medical image computing and computer assisted intervention–MICCAI 2022: 25th international conference, Singapore, September 18–22, 2022, Proceedings, Part VIII (pp. 35 – 45 ). 10.1007/978-3-031-16452-1_4

[b43] Xia , T. , Chartsias , A. , Wang , C. , Tsaftaris , S. A. , & Initiative , A. D. N. ( 2021 ). Learning to synthesise the ageing brain without longitudinal data . Medical Image Analysis , 73 , 102169 . 10.1016/j.media.2021.102169 34311421

[b44] Xie , Y. , & Li , Q. ( 2022 ). Measurement-conditioned denoising diffusion probabilistic model for under-sampled medical image reconstruction . In Medical image computing and computer assisted intervention–MICCAI 2022: 25th international conference, Singapore, September 18–22, 2022, Proceedings, Part VI (pp. 655 – 664 ). 10.1007/978-3-031-16446-0_62

[b45] Yang , L. , Zhang , Z. , Song , Y. , Hong , S. , Xu , R. , Zhao , Y. , Zhang , W. , Cui , B. , & Yang , M.-H. ( 2023 ). Diffusion models: A comprehensive survey of methods and applications . ACM Computing Surveys , 56 ( 4 ), 1 – 39 . 10.1145/3626235

[b46] Zhai , X. , Kolesnikov , A. , Houlsby , N. , & Beyer , L. ( 2022 ). Scaling vision transformers . In Proceedings of the IEEE/CVF conference on computer vision and pattern recognition (pp. 1204 – 1213 ). 10.1109/cvpr52688.2022.01179

[b47] Zhao , Y. , Dong , Q. , Chen , H. , Iraji , A. , Li , Y. , Makkie , M. , Kou , Z. , & Liu , T. ( 2017 ). Constructing fine-granularity functional brain network atlases via deep convolutional autoencoder . Medical Image Analysis , 42 , 200 – 211 . 10.1016/j.media.2017.08.005 28843214 PMC5654647

[b48] Zhu , X. , Thung , K.-H. , Adeli , E. , Zhang , Y. , & Shen , D. ( 2017 ). Maximum mean discrepancy based multiple kernel learning for incomplete multimodality neuroimaging data . In Medical image computing and computer assisted intervention- MICCAI 2017: 20th international conference, Quebec City, QC, Canada, September 11–13, 2017, Proceedings, Part III (pp. 72 – 80 ). 10.1007/978-3-319-66179-7_9 PMC5790115 29392246

