# Supplementary material for: ReMiND: Recovery of missing neuroimaging using diffusion models with application to Alzheimer’s disease
Source: Imaging Neurosci (Camb). 2024 Oct 22;2:imag-2-00323. doi: 10.1162/imag_a_00323 (PMC12290738; doi:10.1162/imag_a_00323)
Supplement: Supplementary Material [file imag_a_00323-supp.pdf]

Supplementary Material

A comprehensive introduction to the ReMiND architecture (Figure 1) is provided below:

- **Fully-Connected (FC) Layer.** A fully-connected layer (or dense layer), is a fundamental component of deep neural networks where each neuron is connected to every neuron in the previous layer. This layer is implemented through matrix multiplication, allowing for efficient computation of the weighted sum of inputs. Fully-connected layers are often paired with non-linear activation functions, enabling the network to learn and represent complex patterns in the data. In ReMiND, the FC layer will take the timestep as the condition, mapping it into a time embedding that is conditioned by the diffusion and denoising process of the diffusion model.

- **Group Normalization Layer.** Group normalization (Wu & He (2018)) is a technique used to normalize inputs across a group of channels, rather than the entire batch, which helps stabilize training in deep neural networks. This method is particularly effective in small batch sizes and improves model performance by reducing internal covariate shifts. In ReMiND, group normalization is utilized for the diffusion process. It processes the visits by dividing the channels into smaller groups, normalizing each group independently to maintain stable activations.

- **Convolution Layer.** A convolutional layer is a key component of convolutional neural networks (CNNs) that applies a set of filters to input data to extract spatial features, such as edges and textures. In ReMiND, by performing convolution operations, this layer preserves the spatial relationships within the visits, making it highly effective for feature extraction in MRIs.

- **Shortcut Connection.** A shortcut connection (or skip connection), is a feature in neural networks that allows the input to bypass one or more layers and be added directly to the output (He et al. (2015)). In ReMiND, the shortcut connection will directly pass the visit to a later layer in the network, enabling the model to alleviate the vanishing gradient problem. This technique helps to mitigate the vanishing gradient problem and enables the training of much deeper networks by facilitating the flow of information and gradients through the network. **Residual Block** is a building block of deep neural networks that includes shortcut connections allowing the input to bypass one or more layers and be directly added to the output.

- **Concatenate Operation.** The concatenate operation in neural networks combines multiple input tensors along a specified dimension, allowing for the merging of features from different layers or branches to enrich the model's representation. In our case, the concatenate operation takes the past visit, current visit, and following visit as inputs, and combines these visits into a unified tensor. This tensor will then be processed by the diffusion model. In ReMiND, concatenate operation binds the past and following visits with the current visit, making the generation conditioned on the past and following visits.

Table 1. Comparison of model performance averaged across 10 test sets. Performance was measured with structural similarity index (SSIM) and peak signal-to-noise ratio (PSNR) in decibels (dB) on the generated MRI images with skull voxels included in the calculation. Performance was evaluated overall and separately by clinical group (CN, MCI, and AD). P indicates the imputation method conditioned on the most recent past image. PF indicates the imputation method conditioned on both the most recent past image and the closest following image. AE indicates imputation using an autoencoder. Bold values indicate the best performing imputation method for a given clinical diagnosis group within the P or PF condition.

Model	Images	CN	MCI	AD	ALL
SSIM (higher=better)					
Naive-P	w/ skull	0.705 ± 0.032	0.710 ± 0.013	0.747 ± 0.026	0.714 ± 0.015
AE-P	w/ skull	0.730 ± 0.010	0.725 ± 0.015	0.735 ± 0.017	0.728 ± 0.011
ReMiND-P	w/ skull	**0.850 ± 0.010**	**0.848 ± 0.006**	**0.850 ± 0.011**	**0.850 ± 0.007**
Naive-PF	w/ skull	0.702 ± 0.061	0.695 ± 0.026	0.707 ± 0.016	0.701 ± 0.014
AE-PF	w/ skull	0.728 ± 0.006	0.739 ± 0.018	0.746 ± 0.016	0.737 ± 0.013
ReMiND-PF	w/ skull	**0.886 ± 0.010**	**0.899 ± 0.010**	**0.900 ± 0.004**	**0.895 ± 0.002**
PSNR (dB) (higher=better)					
Naive-P	w/ skull	22.169 ± 1.253	22.364 ± 0.551	23.704 ± 1.413	22.503 ± 0.692
AE-P	w/ skull	25.013 ± 0.274	24.630 ± 0.442	24.837 ± 0.617	24.780 ± 0.319
ReMiND-P	w/ skull	**27.182 ± 0.549**	**26.939 ± 0.171**	**27.455 ± 0.966**	**27.101 ± 0.285**
Naive-PF	w/ skull	22.241 ± 2.691	22.112 ± 0.851	22.267 ± 0.280	22.255 ± 0.682
AE-PF	w/ skull	24.805 ± 0.227	24.907 ± 0.343	25.002 ± 0.139	24.890 ± 0.273
ReMiND-PF	w/ skull	**29.192 ± 0.608**	**28.870 ± 0.455**	**28.819 ± 0.833**	**28.956 ± 0.406**

Table 2. The 28 regions of the brain.

Amygdala	Pallidum
Caudal Anterior Cingulate	Paracentral
Caudal Middle Frontal	Parahippocampal
Entorhinal	Pars Orbitalis
Fourth Ventricle	Pars Triangularis
Hippocampus	Posterior Cingulate
Inferior Lateral Ventricle	Rostral Anterior Cingulate
Inferior Parietal	Rostral Middle Frontal
Inferior temporal	Superior Frontal
Insula	Superior Parietal
Isthmus Cingulate	Superior Temporal
Lateral Ventricle	Thalamus
Medial Orbitofrontal	Third Ventricle
Middle Temporal	Transverse Temporal

Table 3. Comparison of average progression rates within the CN, MCI, and AD groups. Difference indicates the difference of progression rates between CN and AD groups. Results were averaged across the 10 test sets and averaged across all regions defined by the MNI atlas for ANTs and Aseg atlas for FreeSurfer pipeples. Bold values indicate the best performing imputation method within the P or PF condition.

Stage	Naive-P	AE-P	ReMiND-P	Naive-PF	AE-PF	ReMiND-PF
FreeSurfer						
CN	-	0.096 ± 0.001	0.127 ± 0.009	0.182 ± 0.007	0.082 ± 0.002	0.087 ± 0.002
MCI	-	0.103 ± 0.001	0.140 ± 0.000	0.184 ± 0.002	0.083 ± 0.003	0.096 ± 0.001
AD	-	0.111 ± 0.003	0.143 ± 0.003	0.185 ± 0.002	0.091 ± 0.003	0.103 ± 0.003
**Difference**	-	0.015 ± 0.001	**0.016 ± 0.005**	0.003 ± 0.008	0.009 ± 0.006	**0.016 ± 0.002**
ANTs						
CN	-	0.026 ± 0.006	0.031 ± 0.004	0.020 ± 0.001	0.034 ± 0.001	0.021 ± 0.001
MCI	-	0.038 ± 0.003	0.043 ± 0.004	0.023 ± 0.001	0.036 ± 0.001	0.023 ± 0.001
AD	-	0.036 ± 0.003	0.045 ± 0.001	0.024 ± 0.001	0.038 ± 0.001	0.036 ± 0.001
**Difference**	-	0.010 ± 0.005	**0.014 ± 0.003**	0.004 ± 0.008	0.004 ± 0.006	**0.015 ± 0.001**

Table 4. Comparison of atrophy rate estimation with and without missing data imputation. The atrophy rate was calculated for hippocampus region, parahippocampal region, and the third ventricle. Complete indicates all participants have complete data and serve as the ground truth. With imputation indicates the random half of the participants who have missing data have images imputed using the ReMiND models. Without imputation indicates we do not impute images for participants who have missing data and calculate the atrophy rate using only the half without missing images. The results were averaged across 10 test sets.

Settings	Hippocampus	Parahippocampal	Third Ventricle
Complete	0.0565 ± 0.011	0.0239 ± 0.008	0.1295 ± 0.006
With Imputation	0.0573 ± 0.014	0.0245 ± 0.010	0.1322 ± 0.010
Without Imputation	0.0491 ± 0.013	0.0209 ± 0.009	0.1075 ± 0.007

References

He, K., Zhang, X., Ren, S., & Sun, J. (2015). Deep residual learning for image recognition. *2016 IEEE Conference on Computer Vision and Pattern Recognition (CVPR)*, 770-778. Retrieved from `https://api.semanticscholar.org/CorpusID:206594692`

Wu, Y., & He, K. (2018). Group normalization. *International Journal of Computer Vision*, *128*, 742 - 755. Retrieved from `https://api.semanticscholar.org/CorpusID:4076251`
